# A four-way patient search method for the retrospective identification of poisoning patients

**DOI:** 10.1038/s41598-024-52358-z

**Published:** 2024-01-20

**Authors:** Veronika Uslin, Ville Hällberg, Timo Lukkarinen, Marjo Niskanen, Teemu Koivistoinen, Ari Palomäki

**Affiliations:** 1 Department of Medicine and Surgery, Humanitas University, Via Rita Levi Montalcini 4, Pieve Emanuele, 20072 Milan, Italy; 2 Emergency Department, Kanta-Häme Central Hospital, 13530 Hämeenlinna, Finland; 3 City of Helsinki, Social Services, Health Care and Rescue Services Division, 00100 Helsinki, Finland; 4 Tampere University Hospital, 33520 Tampere, Finland; 5 Faculty of Medicine and Health Technology, Tampere University, 33520 Tampere, Finland

**Keywords:** Data acquisition, Databases, Health care, Addiction

## Abstract

When studying emergency department (ED) visits, electronic health record systems of hospitals provide a good basis for retrospective studies. However, many intoxication patients presenting to the ED, may not be identified retrospectively if only a single search method is applied. In this study, a new four-way combined patient search method was used to retrospectively identify intoxication patients presenting to the ED. The search included reason for admission to the ED, laboratory results related to intoxication diagnostics, ICD-10 codes, and a novel free word search (FWS) of patient records. After the automated search, the researcher read the medical records of potential substance abuse patients to form comprehensive profiles and remove irrelevant cases. The addition of a free word search identified 36% more substance abuse patients than the combination of the other three methods mentioned above. Patients identified by the FWS search alone were generally admitted to the ED for trauma or mental health problems and were often found to be heavily under the influence of alcohol and/or drugs. The main intoxicants were ethanol and benzodiazepines. The free word search was highly complementary to traditional patient search methods, highlighting the importance of the combined patient search method in retrospective data collection.

## Introduction

Acute poisonings, both accidental and deliberate, pose a significant strain on emergency departments (EDs) around the world^1,2^. According to a recent estimate, over 96 million or 29% of people aged 15–64 living in the European Union have experimented with illegal substances^3^. While availability and fashion shape the illegal drug market, burdensome times, like war or the Covid-19 era, create anxiety. The latter can be seen e.g. in mentally unstable persons’ attendance at the ED due to intoxication^4^. The consensus is that intoxication cases due to both legal substances, such as ethanol, and illegal substances are on the rise^3,5,6^.

Intoxicated patients arriving at the ED are often in a critical condition and short-term mortality is high after acute poisoning due to substance abuse^7,8^. When the patient may not be able to provide enough information or, in the case of illicit substances, may not want to, accurate diagnosis for proper treatment is difficult. Therefore, it is essential to keep track of the constant change in the healthcare setting, while not forgetting cultural differences. In Europe, ethanol is the leading cause of attending the ED due to intoxication for an intoxication visit^2,5,9^, whereas in Saudi Arabia ethanol related cases are virtually unknown, the most commonly abused substance being paracetamol^10^. Pesticide poisonings, such as organophosphate, are more common in developing countries^11–13^. The range of substances available is also diverse, as drug content often varies from shipment to shipment and new drugs, such as novel psychoactive substances (NPS), appear in rapid speed^3^.

Only a small number of studies on the flow of poisoning patients in ED have been carried out in Europe during the last two decades. Many of these studies were conducted retrospectively using only one data abstraction method [2, 5, 9, Additional Table 1]. In this study, our primary aim is to create a precise method for retrospective intoxication data search. We present our four-way combined patient search method and its performance. Additionally, the results of a pilot analysis on 131 patients are reported.

## Methods

### Study setting

Kanta-Häme Central Hospital (KHCH) is a secondary care hospital with a catchment population of 171,000. It is located in the city of Hämeenlinna, Southern Finland, where the geographical centre of the Finnish population is^14^. The ED provides emergency care on average for 45,800 patients annually. The collected data consists of patients (≥ 18 years) treated in the KHCH ED for acute poisoning. Our research consists of data collected prior to the shutdown caused by the Covid-19 pandemic, and is therefore assumed to reflect average acute poisoning cases and to include typical seasonal variations^4,15^. In this pilot analysis, we report our combined search method and the results of the sample of the patients born on the first four days of each month.

### Data collection

The electronic health record (EHR) system used in Kanta-Häme Wellbeing Services County is called Lifecare™ (Tietoevry Oy, Espoo, Finland). It consists of data produced both in public primary and secondary healthcare. Lifecare™ is a moderately structural system, with a large amount of information stored in a completely structured manner (laboratory results, diagnoses, medication). However, most of the data are gathered and stored as free text, but under different headings and sub-headings, depending on the context.

Lifecare™ runs on Structured Query Language (SQL) server database, which is completely copied daily for statistical and management purposes to a statistical server. Apart from the laboratory data, the data were collected with Microsoft SQL server management tools from the statistical server and converted to Microsoft Excel in CSV-format. All data collection were limited within SQL code identifying the patients attending the KHCH emergency department 1.1.2019 to 31.12.2019. Laboratory data were received from a third-party company Fimlab Oy (Tampere, Finland) that analyses KHCH’s patient samples.

The EHR system and laboratory data were screened with the specifications described here.

1. Reason for attending was searched from the physician’s referral or determined by an attending triage nurse, whichever applied. Keywords used for the search were *intoxication, intoxicant, impaired consciousness, unconsciousness, convulsion, drug* and *substance abuse.*
2. Laboratory data were received from the Fimlab database. Blood and urine samples from the year 2019 were searched for the mention of illicit substances, ethanol, and certain pharmaceuticals, such as antidepressants and paracetamol (Additional Table 2).
3. Discharge diagnoses for both inpatients and outpatients were searched according to ICD-10 codes (Table 1).
4. A free word search for all 45,152 patient visits in the ED in 2019 was carried out with SQL code as follows (Table 2).
  - All EHR charts under General Practice, Surgery, Internal Medicine, Pulmonology and Neurology together with possible external referrals were searched for each of the words listed (Table 2). All words were in Finnish and in their basic grammatical form.
  - If any of the words matched with any of the EHR charts, with all subheadings included, the EHR chart was requested as full text for further analysis. Altogether 260 378 charts were screened.

**Table 1:** ICD-10 codes used in data search.

ICD-10 codes (n = 43)	
Alcohol related MBD	F10
Drug related MBD	F11–F16, F19
Smoke exposure	X00–X09
Accidental poisoning	X44–X49
Self-harm via poisoning	X69
Poisoning	T36#, T40
Undetermined poisoning	T50
Toxic effect of a substance	T51–T65

*MBD* Mental and behavioral disorder. A total of 43 ICD-10 codes thought to indicate intoxication were searched from the EHR. The codes were used as discharge diagnoses and marked by a clinician.

**Table 2:** Free word search to patient report.

Search words (n = 27)	
Intox*	Blood test for poison
Overdose	Self-harm
Convuls*	Benzodiazepin*
Unconscious*	Amphetamine
Poison	Gamma
Drug	Cocain*
Drunk	Cannabi*
Alcohol	Opiates
Per mille	Hypoglycem*
Substance	Fire

In the free word search, 20 main concepts were searched from EHR and are shown on the table. They were used in their most basic form in Finnish language (*). Considering different conjugations, a total of 27 words were searched. “Gamma” refers to Gamma-butyrolactone (GBL) or Gamma-hydroxybutyrate (GHB). The data collection was possible with SQL-code.

We considered that if any keyword was found in any of the four different searches listed above, there was a clinical suspicion of poisoning. After this step, the electronic health records of each individual patient (identified as a potential substance abuser) were manually checked to determine whether or not they met the criteria for intoxication. In addition to a history of substance use, laboratory tests and possible blood alcohol levels, signs and symptoms of intoxication were also verified. The latter were particularly required in the case of alcohol intoxication.

In cases where the in-hospital EHR was lacking blood alcohol concentration data, possible paramedic reports were also read. Psychiatric EHR was accessed if mental health was a contributor to the intoxication visit. When reviewing the EHR for paramedic, triage and hospital data, the cases were judged to either meet the criteria of acute poisoning or not. Overlapping of the data was controlled according to individual social security number (SSN) and the date of attending the visit.

### Review of electronic health records

Substances used and necessitating the visit were collected from patient history and laboratory findings. In the case of multiple substances, the most significant intoxicant regarding its toxicity and correlation to symptoms described in the patient report was noted as the primary intoxicant. Responses to certain treatments were noted, such as response to flumazenil when benzodiazepine intoxication was suspected. In case of a positive reaction to an antidote, the substance was considered to have been used. Substances marked as secondary contributed to the state of intoxication.

When acute alcohol intoxication (AAI) was suspected in the case of clinically significant behavioural or psychological changes^16^, either an alcohol breath test or a blood sample was taken. The AAI was further divided into three categories depending on the blood alcohol concentration (BAC): mild AAI (BAC 10.9–21.6 mmol/l, 0.50–0.99 per mille), moderate AAI (BAC 21.7–65.0 mmol/l, 1.00–2.99 per mille) and severe AAI (BAC > 65.1 mmol/L, ≥ 3.00 per mille). The definition of severe AAI is based on increased risk of respiratory failure^17^.

In addition to the patient’s account, chronic substance use was considered in terms of comorbidity, e.g., when the patient had alcohol-related pancreatitis and possible multiple ED visits due to intoxication. Diagnoses of chronic or long-lasting somatic and mental diseases were collected according to the ICD-10 codes. If the diagnosis was not marked down at the index visit, those of the two preceding years were considered still active and were included.

The acute reasons for the intoxication were divided into three groups: accidental, recreational substance abuse, and deliberate self-harm. Accidents were defined as unintentional consumption, such as carbon monoxide poisoning during a fire, or the patient having ingested excessive medication due to dementia. Recreational substance abuse included unintentional alcohol or drug overdoses for non-medical purposes and traumas occurring under the influence of intoxicants. Self-harm was defined as histrionic substance abuse to attract attention, suicidal ideation or suicide attempts.

The study has the institutional approval of the Wellbeing Services County of Kanta-Häme [KHSHP/233/13.00.01/2022]. According to the Finnish legislation, register studies in which subjects are not contacted or subjected to any intervention do not require the approval of the Ethics Committee. Nor is informed consent required. All methods were carried out in accordance with relevant guidelines and regulations.

### Statistics

Statistical analysis was carried out using IBM Statistical Package for Social Sciences (SPSS) for Windows, version 28. Dichotomous variables were compared with Pearson Chi-Square test. Statistical significance was set at *p* < 0.05.

## Results

### The combined search

**Figure 1:**
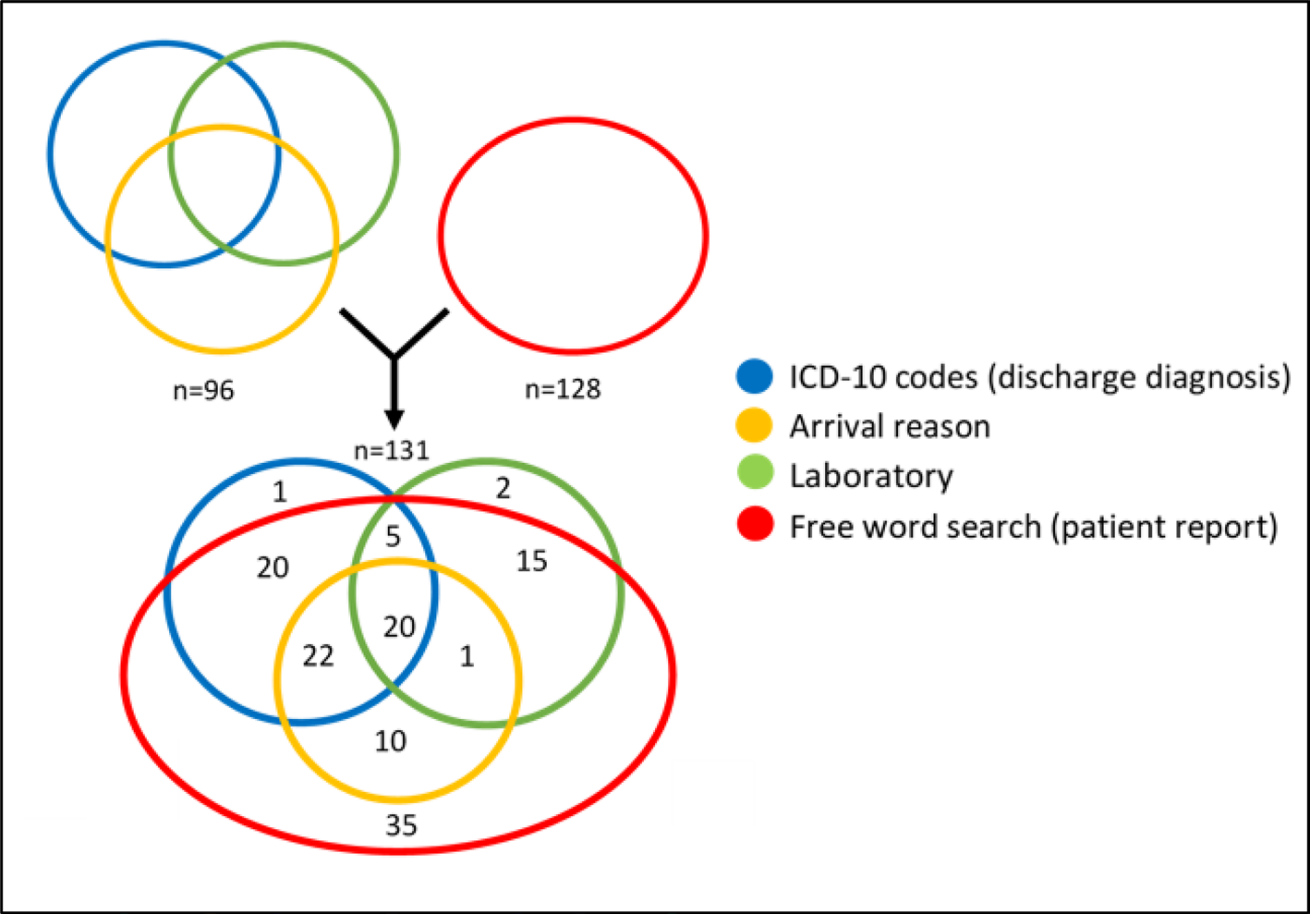
Patients found using four search methods. Blue: ICD-10 codes, Green: Laboratory search, Yellow: Reason for attending. Red: Free word search. The patients from the first four days of each month formed the study population and comprised 131 patients with 165 visits.

The first part of the study, including patients born on the first four days of each month, who attended the ED, utilized previously applied search methods (reason for attending, laboratory results, and ICD-10 codes). This search yielded 222 potential intoxication visits. After reading each suspected intoxication patient’s EHR, 96 patients with 117 visits were found. A second round with a free word search yielded 281 potential intoxication visits. The in-depth review of EHRs yielded 128 intoxication patients and 162 visits. There were 35 new intoxication patients and 48 visits which were not found during the initial searches (Fig. 1), showing a 36% increase in the number of patients. On the other hand, three patients were discovered only through ICD-10 code or laboratory result search. When all the four searches were combined, a total of 131 patients with 165 visits resulted after the reviews of the individual EHRs.

### Patients found by one method only

An analysis of patients who were found only by FWS included 35 patients with 48 visits, 39 visits involving alcohol only, one visit involving both alcohol and drugs and eight visits involving drugs and/or illicit substances. The most common reason for admission was head injury or other trauma in 28 visits and the second most common reason was mental health problems in 9 visits. Among patients for whom alcohol was the primary intoxicant, the median alcohol breath test was 1.8 per mille (range 0.58–2.78) for trauma patients and 2.34 per mille (1.1–3.8) for mental health patients. Four visits ended because the patient left without being seen. They had alcohol breath test results ranging from 1.69 to 3.8 per mille on admission. These patients had a history of heavy and long-term alcohol, excessive drug and/or illicit substance use.

We had also three patients who were not found by FWS. Laboratory tests revealed amphetamine, cannabinoids and benzodiazepines in one patient with a diagnosis of undetermined confusion, and a blood alcohol level of 1.57 per mille in one trauma patient. These findings are likely to have had a major impact on the condition of these patients. One patients whose visit was found only by ICD-10 code had a snakebite.

### Profile of intoxication patients

The profiles of intoxication patients and intoxications are presented in Table 3. For men, the mean age was 44.4 years (SD = 17.3) and for women 50.7 years (SD = 20). The proportion of self-harm was more than threefold in women compared to that in men (*p* < 0.001). Men had significantly more substance abuse than women (*p* < 0.001). About a quarter of the patients had two or more intoxication vistis during one-year follow-up. More than a third of these patients had a diagnosis related to mental health (Table 3).

**Table 3:** Profiles of the intoxication patients.

	Total n (%)	Men n (%)	Women n (%)	Overall *p*-value
Number of patients	131	94 (72)	37 (28)	*p* < 0.001
Number of visits	165	110 (67)	55 (33)	N.S.
Median age (IQR)	46.6	44.4 (30–59)	50.7 (33–69)	N.S.
Reason for intoxication (n = 165)				< 0.001
Accident	6 (4)	4 (4)	2 (4)	
Substance abuse	124 (75)	93 (84)*	31 (56)*	
Self-harm	35(21)	13 (12)*	22 (40)*	
Recurrence (n = 131)				N.S.
One visit (index)	99 (76)	75 (80)	24 (65)	
Two or more visits	32 (24)	19 (20)	13 (35)	
Concomitant problems (n = 131)				N.S.
Mental health disorder	48 (37)	30 (32)	18 (49)	
Chronic substance abuse	70 (53)	50 (53)	20 (54)	
Somatic disease	77 (59)	51 (54)	26 (70)	

*IQR* Interquartile range. Number of acute intoxication visits of patients born in the first four days of every month to the ED in 2019. The index visit was the first intoxication episode of the patient in 2019. A patient may have had none, one or more than one concomitant diagnoses.

*p-value < 0.001 between genders in Abuse and Self-harm.

### Intoxicants

The most common intoxicant was ethanol, followed by benzodiazapines and other pharmaceuticals. In at least one quarter of the visits, the use of more than one substance was observed (Table 4). Ethanol was observed in 122 visits, being the primary intoxicant in 111 cases (67%). The median for ethanol BAC was 43.6 mmol/L (2.01 per mille, moderate AAI) for men and 31.2 mmol/L (1.44 per mille, moderate AAI) for women. There were 13 severe AAI cases (BAC > 65.1 mmol/L, 3.00 per mille).

**Table 4:** Intoxicants.

Substance (n = 165)	Primary n (%)	Secondary n (%)	Total n (%)
Ethanol	111 (67)	11 (7)	122 (74)
Benzodiazapines	16 (10)	9 (5)	25 (15)
Other pharmaceuticals	14 (8)	18 (11)	32 (19)
Cannabinoids	7 (4)	13 (8)	20 (12)
Amphetamine and derivatives	8 (5)	10 (6)	18 (11)
Opioids	3 (2)	2 (1)	5 (3)
Cocaine	1 (< 1)	1 (< 1)	2 (1)
Other	5 (3)	0	5 (3)

Substances used. Benzodiazepines and opioids were excluded from the pharmaceuticals to highlight their usage at acute intoxication visits. Acute intoxications labelled as “other” included carbon monoxide poisoning, animal and plant toxins. “Amphetamine and derivatives” entail methamphetamine and MDMA. “Other Pharmaceuticals” included paracetamol, insulin, pregabalin, antidepressants (tricyclic, SSRI/SNRI), antipsychotics, anticonvulsants, beta-blockers, NSAIDs.

## Discussion

When improving the retrospective data search from the hospital’s EHR system, free word search added more than one-third intoxication patients to the yields of a combination of three established methods: reason for attending, laboratory findings, and ICD-10 diagnosis. In the analysis of the pilot material, the vast majority of the intoxication patients were men. Ethanol was detected in three quarters of the visits. Benzodiazepines were the most common drug while cannabinoids and amphetamine were the most common illegal substances. Additionally, half of the women and one third of the men had a mental health related diagnosis.

### The combined search

We conducted a PubMed search with the search words (*emergency* AND [*department* OR *room*]) OR *casualty* combined with *acute poisoning* OR *accidental poisoning* OR *intoxication* OR *suicide* OR *self-poisoning* OR *recreational drug* OR *poison* for the period 1.1.2000–31.12.2022. Different data abstraction methods for intoxication patients were found in this search and in other relevant articles (Additional Table 1). First, in a couple of studies, reasons for attending were searched from the EHR^18,19^. Second, laboratory information was used in some recent studies^20–22^. Third, ICD-10 codes from T36 to T65 were used in several studies^2,23–25^. In our study, we used altogether 43 ICD-10 codes, including all these previously mentioned codes and several additional F and X diagnose codes concerning alcohol and substance misuse. In some of the earlier studies, only the hospital’s own medical register or the national health database was mentioned as the source material but no detailed data abstraction method was presented.^9,12,26–28^.

Single word search for “paracetamol” in the diagnostic area for the ED report was used in one study, while in another study, a four-word search, “poisoning, alcohol, CO and/or organophosphates” was used^29,30^. In our study, which was conducted using SQL-based free word abstraction for all patients visited the ED in 2019, the search query included 27 words. One study used a combination of word search and ICD-10-AM codes (Australian Modification) to find opioid intoxications from a compact data set. The word search was conducted for a specific variable describing the events of the visit^31^. This data abstraction method was closest to ours.

We found few observational/prospective studies^5,32^. Conducting a prospective study requires extensive organization, staff commitment, and avoidance of biases. Additionally, the patient’s permission is needed as the data are collected solely for research purposes. As reported by the researchers, these aspects may constrain the size of the data^5^.

### FWS versus traditional search methods

In this study, a significant proportion of patients were found only using the FWS method and a few patients using one of the other search methods.

Patients found using FWS alone had come to the ED primarily for reasons other than poisoning. Their alcohol and drug tests showed that they mostly had high alcohol levels or mixed use of several drugs. Additionally, based on the symptoms and signs, intoxicants had contributed substantially to the admission to the ED. The lower alcohol levels in our study certainly do not meet the traditional hallmarks of alcohol intoxication. However, since all these patients had a history of alcohol and/or drug abuse and often several hours had passed from usage by the time the patients arrived to the ED, we considered their results to be potentially significant. For a few patients, we considered that even relatively low alcohol levels supported the diagnosis of alcohol intoxication because of the typical symptoms and lack of information on other intoxicants.

The diversity of language used in the free-form medical record text limits the sensitivity of the usually functional FWS system. As a result, we continue to consider laboratory search and ICD coding as necessary complementary search methods.

An article by Kaji^33^, discusses in detail the guidelines for retrospective studies. One of the major problems is bias due to insufficient material extraction. In the present retrospective pilot analysis, we tried to avoid these obstacles by using a novel four-way data extraction method.

### Patient profile and substances

Culture, socioeconomic status, and population structure in the country of study influence many aspects of intoxication, among them the intention, gender and age distribution^34,35^. In developed countries, a typical intoxication patient is a young to a middle-aged man^2,5,20^. In developing countries, the majority of intoxication patients are younger^11–13,30,36^ and often over half are women^12,13,30,36^.

In earlier studies, the reason for attending the ED due to intoxication was most often substance abuse in developed countries, as it was in our study^2,5,9^. Our finding that women have a higher incidence than men of intoxication cases involving self-harm is in accordance with earlier studies^2,10,13,36^. One explanation for this higher incidence may be the cultural pressure and domestic stress to which many women are subjected^13^.

As in our study, ethanol has been the most common substance in many intoxication studies, frequently followed by benzodiazepines^2,9,20^. In some studies, opioid use was more common^2,37^, whereas in our material this was rarely detected. Pharmaceutical misuse constitutes major problem among ED patients^38^, which was also seen in our patients. Amphetamine, and its derivatives (methamphetamine), were identified more often and cocaine was rare when compared to several other studies^2,9,20^. Our findings concur with the findings of wastewater analyses conducted in Finland^39^.

Polysubstance misuse was detected in 12–31% of the visits, the leading non-alcoholic substances being benzodiazepines or opiates^2,5,37^. Our findings were similar to those of earlier reports, since at least a quarter of our patients were under the influence of multiple substances. Alcohol was often combined with benzodiazepines, amphetamine, and cannabinoids.

In earlier studies, recurring emergency visits due to intoxications have been detected in 5–12% of patients in up to one year of follow-up^2,9,20^. We found that a quarter of the patients had at least one repeat intoxication visit during one year. This higher frequency of attending the ED may be due to the exact one-year follow-up and the inclusion of chronic alcohol misuse. Overall, we considered that in half of our patients the substance use was continuous.

### Strengths and limitations

A strength of the present study is that our modern combined four-way search of the hospital EHR system revealed more intoxication patients than a traditional search conducted using single parameter. The EHR system enabled broad, retrospective data collection. The use of the four methods was estimated to gather information on all intoxication patients as completely as possible. For example, if the clinician had treated trauma as a result of intoxication, a single specification like an ICD-10 code or reason for attending related to intoxication may have been missed. In such cases, our combined four-way search was more powerful than the use of a single specification like ICD-10 code.

We chose to gather our retrospective data from 2019. Since all the intoxication visits studied took place prior to the outbreak of Covid-19 in Europe, the pandemic did not influence our results. For the pilot analysis, we included data on the patients born on the first four days of each month. The strength is that the patient data were presumably not skewed in any sense.

After the combined four-way search, our pilot analysis of intoxications included less than two hundred cases, which could be considered a limitation. The material was collected retrospectively, which may have caused some data to be missed. For example, notation differences or misspellings may have affected data obtained from the EHR.

Laboratory results also have their limitations and may lead to misdiagnosis. Cross-reactivity is very common with amphetamines, e.g. with bupropion or ranitidine. In these cases medical records were often helpful, but there are also substances that were not detected by traditional laboratory methods like gamma-hydroxybutyrate or gamma-butyrolactone, and can only be found based on clinical suspicion and history.

Another limitation of our research is our definition of alcohol intoxication. Differences in individual alcohol tolerance have a significant impact on the symptoms of alcohol use. Sometimes a patient may also present to the ED with significant symptoms hours after consuming alcohol, but the alcohol level at this point will only be less than one per mille. Hence, the distinction between poisoning and moderate intoxication is sometimes unclear. Because our patients required monitoring and treatment in the ED, the terminology may not be relevant to patient flow.

As this was a single centre study, the results may not be widely generalizable.

## Conclusion

A novel four-way combined patient search method revealed more intoxication patients than a traditional retrospective search. A free word search in the EHR system of the hospital revealed over one third more patients than the combined search of reason for attending, laboratory findings, and ICD-10 codes. In the analysis of our pilot patient population, ethanol was the main substance ingested and causing intoxication. Misuse of prescription drugs was a notable problem, which should be better analysed later. With our modern data abstraction method, we hope to achieve a comprehensive picture of acute intoxication patients.

### Supplementary Information

Supplementary Tables.

## Data Availability

The data that supports the findings of the study are available from the corresponding author upon reasonable request and with the permission of Well-being Services County of Kanta-Häme.
