# Supplementary material for: A four-way patient search method for the retrospective identification of poisoning patients
Source: Sci Rep. 2024 Jan 20;14:1801. doi: 10.1038/s41598-024-52358-z (PMC10799932; doi:10.1038/s41598-024-52358-z)
Supplement: Supplementary file 1 — Supplementary Tables. [file 41598_2024_52358_MOESM1_ESM.docx]

**A four-way patient search method for the retrospective identification of poisoning patients**

Veronika Uslin

Ville Hällberg

Timo Lukkarinen

Marjo Niskanen

Teemu Koivistoinen

Ari Palomäki

**Additional tables 1. and 2.**

**Additional Table 1.** References according to patient search method. Abbreviations: 0 = search method not used, 1 = search method used, NA = not applicable. Number of references using that patient search method in brackets in the title bar.

| Ref. No | Arrival reason  (2 ref.) | ICD-10 or related  (12 ref.) | Laboratory analysis  (3 ref.) | Word search  (3 ref.) | Observational  (5 ref.) | Not specified or not electronic  (9 ref.) |
| --- | --- | --- | --- | --- | --- | --- |
| 1 | 0 | 1 | 0 | 0 | 0 | 0 |
| 2 | 0 | 1 | 0 | 0 | 0 | 0 |
| 3 | NA |  |  |  |  |  |
| 4 | 0 | 1 | 0 | 0 | 0 | 0 |
| 5 | 0 | 0 | 0 | 0 | 1 | 0 |
| 6 | 0 | 0 | 0 | 0 | 0 | 1 |
| 7 | 0 | 1 | 0 | 0 | 0 | 0 |
| 8 | 0 | 0 | 0 | 0 | 1 | 0 |
| 9 | 0 | 0 | 0 | 0 | 0 | 1 |
| 10 | 1 | 0 | 0 | 0 | 0 | 0 |
| 11 | 0 | 0 | 0 | 0 | 1 | 0 |
| 12 | 0 | 0 | 0 | 0 | 0 | 1 |
| 13 | 0 | 0 | 0 | 0 | 0 | 1 |
| 14 | NA |  |  |  |  |  |
| 15 | NA |  |  |  |  |  |
| 16 | NA |  |  |  |  |  |
| 17 | NA |  |  |  |  |  |
| 18 | 1 | 0 | 0 | 0 | 0 | 0 |
| 19 | 1 | 0 | 0 | 0 | 0 | 0 |
| 20 | 0 | 0 | 1 | 0 | 0 | 0 |
| 21 | 0 | 0 | 1 | 0 | 0 | 0 |
| 22 | 0 | 0 | 1 | 0 | 0 | 0 |
| 23 | 0 | 1 | 0 | 0 | 0 | 0 |
| 24 | 0 | 1 | 0 | 0 | 0 | 0 |
| 25 | 0 | 1 | 0 | 0 | 0 | 0 |
| 26 | 0 | 0 | 0 | 0 | 0 | 1 |
| 27 | 0 | 0 | 0 | 0 | 0 | 1 |
| 28 | 0 | 1 | 0 | 0 | 0 | 0 |
| 29 | 0 | 0 | 0 | 1 | 0 | 0 |
| 30 | 0 | 0 | 0 | 1 | 0 | 0 |
| 31 | 0 | 1 | 0 | 1 | 0 | 0 |
| 32 | 0 | 0 | 0 | 0 | 1 | 0 |
| 33 | NA |  |  |  |  |  |
| 34 | 0 | 1 | 0 | 0 | 0 | 0 |
| 35 | 0 | 1 | 0 | 0 | 0 | 0 |
| 36 | 0 | 0 | 0 | 0 | 0 | 1 |
| 37 | 0 | 0 | 0 | 0 | 0 | 1 |
| 38 | 0 | 0 | 0 | 0 | 1 | 0 |
| 39 | 0 | 1 | 0 | 0 | 0 | 0 |
| 40 | 0 | 0 | 0 | 0 | 0 | 1 |

**Additional Table 2**. Laboratory tests. Any laboratory tests conducted on patients attending the ED during 2019 were examined. If any of the tests below were positive or if the drug concentration exceeded the reference value, the patient's electronic medical records were checked.

| Search | Method | Substances | Result |
| --- | --- | --- | --- |
| Drug (Rapid test) | Urine, | Amphetamine | Positive OR Negative |
|  | immunological | Methamphetamine |  |
|  | screening | Barbiturates |  |
|  |  | Benzodiazepines |  |
|  |  | Cocaine |  |
|  |  | Methadone |  |
|  |  | Opiates |  |
|  |  | Cannabis |  |
|  |  | Tricyclic antidepressants |  |
|  |  | Buprenorphine |  |
|  |  | Norbuprenorphine |  |
|  |  |  |  |
| Drug | Urine, | Amphetamine | Amount in urine |
|  | mass | Benzodiazepines |  |
|  | spectrometry | Buprenorphine |  |
|  |  | Cannabis |  |
|  |  | Cocaine |  |
|  |  | Methadone |  |
|  |  | Opiates |  |
|  |  |  |  |
| Drug | Serum, | Paracetamol | Amount in serum |
|  | enzymatic | Salicylates |  |
|  | screening | Phenytoin |  |
|  |  | Carbamazepine |  |
|  |  | Valproate |  |
|  |  | Digoxin |  |
|  | Colorimetry | Lithium |  |
|  |  |  |  |
| Alcohol | Serum, | Ethanol | Amount in serum |
|  | gas | Methanol |  |
|  | chromatography | Ethylene glycol |  |
|  |  | Isopropyl alcohol |  |
|  |  |  |  |
| Ethanol | Plasma, | Ethanol | Amount in plasma |
|  | enzymatic  screening |  |  |
| Carbon monoxide | Venous blood, | Carbon monoxide | Amount in blood |
|  | gas |  |  |
|  | spectrophotometry |  |  |
